# Axillary Artery Injury Caused by Fracture of Humerus Neck and Its Repair Using Basilic Vein Graft

**DOI:** 10.1155/2014/430583

**Published:** 2014-06-30

**Authors:** Vikas Deep Goyal, Vipin Sharma, Sandeep Kalia, Manik Sehgal

**Affiliations:** ^1^Department of Cardiothoracic and Vascular Surgery, Dr. RPGMC Kangra at Tanda (H.P.), Kangra, Himachal Pradesh 176001, India; ^2^Department of Orthopedics, Dr. RPGMC Kangra at Tanda (H.P.), Kangra, Himachal Pradesh 176001, India

## Abstract

Proximal humerus fractures are rarely associated with axillary artery injury. We present a case of a 59-year-old female who had fracture neck humerus along with absent pulsations in the left upper limb after blunt trauma. Computed tomographic angiogram revealed complete occlusion of the left axillary artery. Urgent surgical intervention was done in the form of fixation of fracture followed by exploration and repair of axillary artery. Axillary artery was contused and totally occluded by fractured edge of humerus. Repair of the axillary artery was done using basilic vein graft harvested through the same incision. Postprocedure pulsations were present in the upper limb.

## 1. Introduction

Injuries of the axillary artery are not common [1, 2] and fractures of the upper end of the humerus/humerus neck are rarely associated with injuries of the axillary artery [3, 4], probably due to the abundance of loose connective tissue and soft tissue space in the axilla along with absence of tight compartments. Fractures of the distal one-third of the humerus in contrast to the proximal humerus are commonly associated with brachial artery injury and radial nerve injuries because of tight compartments and close proximity of the neurovascular bundle with the humerus. Scapular fracture, shoulder dislocation, and fracture clavicle are other injuries which can be associated with axillary artery injuries. Children are more susceptible to vascular injuries of the upper limb, especially supracondylar fracture, than adults.

Penetrating trauma of the upper extremities leading to vascular injuries is more common than blunt injuries in both military and civilian population; the incidence of penetrating trauma in upper limb vascular injuries varies from 60 to 90% as reported in various studies in literature [5, 6]. Gunshot injuries, stab with a sharp weapon, and missile injuries constitute the majority of penetrating injuries. Blunt trauma is not commonly associated with axillary artery injuries [7, 8]; however, fall from height and road traffic accidents can sometimes lead to axillary artery injuries.

Repair of axillary artery can be done either by direct repair or by using grafts like saphenous vein, prosthetic grafts, or basilic vein as was done in this case. Most of the literature on repair of upper limb vascular injuries is either on the use of saphenous vein graft [9] or prosthetic grafts [9] or recent advances like endovascular intervention in the form of stent grafts. Basilic vein has not been commonly used for repair of vascular injuries of the upper limb although it is more commonly used for creation of arteriovenous fistula for dialysis access. There is increasing trend towards endovascular management of vascular injuries, and gunshot injuries of the axillary artery have also been managed using stent graft [10] in stable patients.

## 2. Case Report

**Figure 1:**
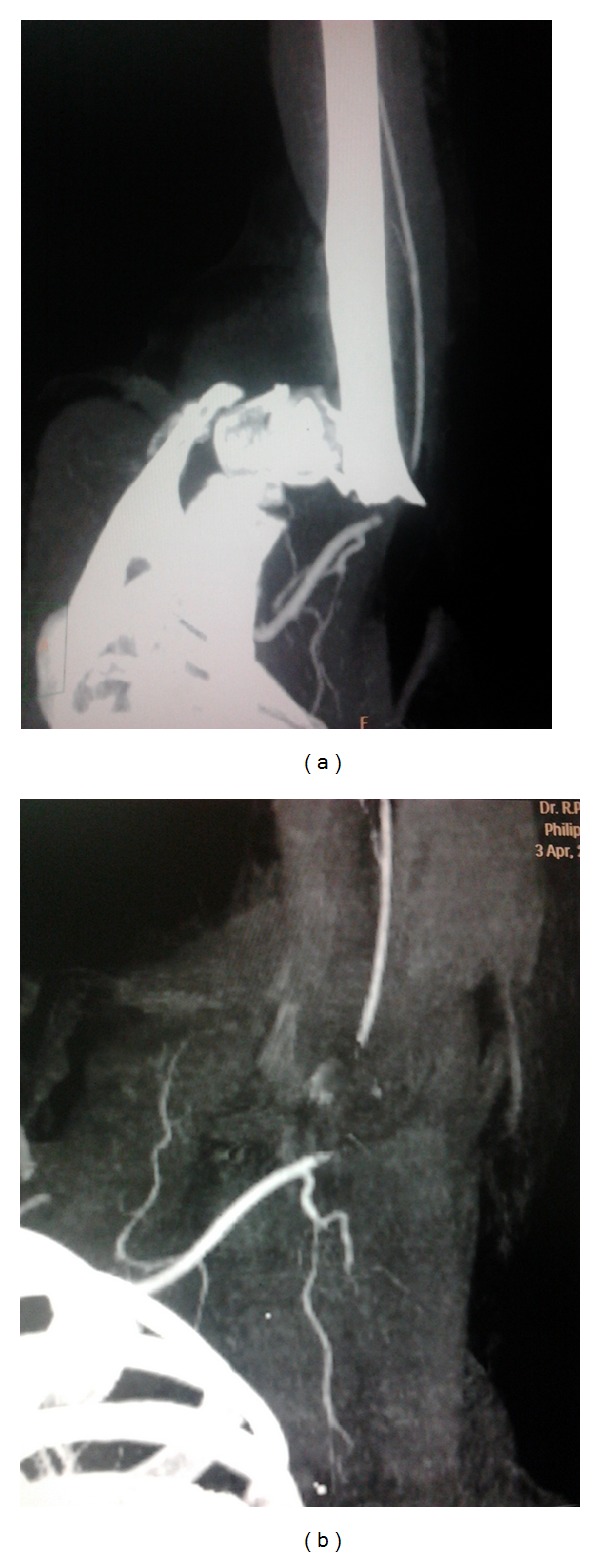
(a) Preoperative CT angiogram showing injury to left axillary artery by fracture proximal end of humerus. (b) Preoperative CT angiogram showing complete occlusion of left axillary artery.

**Figure 2:**
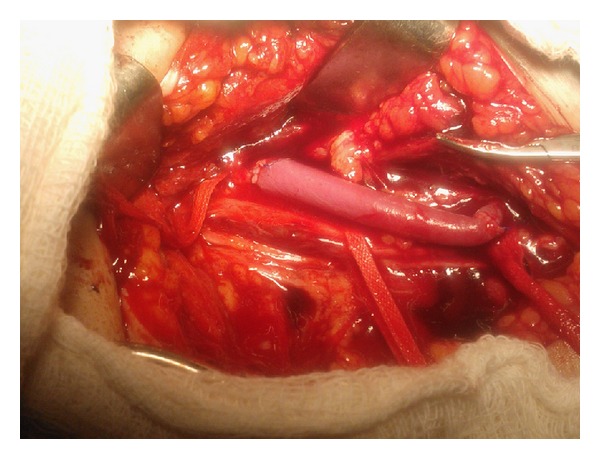
Intraoperative photograph showing repair of axillary artery using reversed basilic vein graft.

A 59-year-old female patient presented with fracture neck humerus along with absent pulsations in the left upper limb due to blunt trauma. Patient also had history of diabetes mellitus and hypertension. Colour Doppler study revealed monophasic flow in the upper limb arteries suggestive of proximal occlusion. Computed tomographic (CT) angiogram showed complete occlusion of the left axillary artery (Figures 1(a) and 1(b)) by sharp edge of the fractured humerus and distal filling of brachial artery through collaterals. Although patient had absent pulsations in the upper limb, capillary refilling was there. Urgent surgical intervention was done in the form of fixation of fracture followed by repair of the axillary artery. Patient was operated under supraclavicular block; orthopedic team first did the open reduction and fixation of the neck of the humerus through a longitudinal incision over the anterolateral aspect of left shoulder. After fixation of fracture, vascular surgery team did the exploration of axillary artery. Axillary artery was explored in the left axilla through a separate incision other than that used for fixation of fracture. Longitudinal incision midway between anterior and posterior axillary folds was given and extended on to the medial aspect of the proximal arm. The axillary artery was found contused for a segment of approximately 8 cm; fortunately, there was no associated nerve injury and the basilic vein was also intact allowing us to use the basilic vein for repair of the axillary artery. Contused segment of the axillary artery was excised after taking proximal and distal control and after heparinization (1 mg/kg). Basilic vein of appropriate length was harvested through the same incision and axillary artery was repaired using reversed basilic vein graft in an end to end fashion using 6-0 polypropylene sutures (Figure 2). Postprocedure pulsations were present in the left upper limb. Fasciotomy was not done as there was no evidence of compartment syndrome and the limb was not edematous. Total duration of procedure including both fixation of fracture and repair of the axillary artery was approximately three hours. Patient recovered well and came for follow-up three months after the procedure with palpable pulsations.

## 3. Discussion

Injuries of the axillary and subclavian artery are associated with high mortality and morbidity rates. High index of suspicion is required for early diagnosis of vascular injuries after proximal fractures of the humerus. Urgent intervention is required for axillary artery injury to prevent excessive hemorrhage or amputation. Associated injuries of the brachial plexus can also complicate the management and require due consideration; fortunately, in this patient, brachial plexus was intact. There are few reports in literature on axillary artery injuries [11] caused by proximal fractures of the humerus and their management using basilic vein graft.

Basilic vein can be a useful graft for upper limb vascular injuries. Advantages of using basilic vein for upper limb vascular injuries are as follows. (1) can be harvested through the same incision, (2) the patient can be operated under regional anesthesia, (3) avoids extra incisions in other limbs, (4) cosmetically better for the patient, (5) easy to harvest, (6) is cost effective and has less chances of infection in comparison to prosthetic grafts. There can also be certain instances where use of basilic vein is either not possible or may not be preferred like in cases where the basilic vein itself is found injured or when there is extensive soft tissue damage or in long segment arterial repairs.

Basilic vein has also been used for arterial reconstruction in the iliofemoral segment [12] and also for repair of popliteal arteries; but the use of basilic vein for repair of arterial injuries of the upper limb [13, 14] is not common, but rather the saphenous vein and prosthetic grafts [15] have been used commonly for vascular repair/reconstruction in the upper limb. Stent grafts have also shown promising results in the management of vascular injuries of the upper limb.

In our experience with repair of brachial artery injuries, we have never used saphenous vein for repair but rather the basilic vein was used every time and was harvested through the same incision and allowed the procedures to be done under regional anesthesia. We are of the opinion that basilic vein is a more suitable and convenient graft for upper limb vascular injuries. Unless the basilic vein itself is damaged by the trauma, it can be conveniently used for upper limb vascular injuries involving axillary and brachial arteries.

## 4. Conclusion

Basilic vein graft can be a suitable alternative in repair of upper limb vascular injuries. Fracture of proximal humerus neck can sometimes lead to axillary artery injury.

## References

[1] Carli A, de Jesus C, Martineau PA (2012). Isolated artery due to blunt trauma in ice hockey. *Clinical Journal of Sport Medicine*.

[2] Oç M, Güvener M, Uçar HI, Akbulut B, Yilmaz M, Ersoy U (2007). Isolated axillary artery injury due to blunt trauma. *Ulusal Travma Ve Acil Cerrahi Dergisi*.

[3] Yagubyan M, Panneton JM (2004). Axillary artery injury from humeral neck fracture: a rare but disabling traumatic event. *Vascular and Endovascular Surgery*.

[4] Matheï J, Depuydt P, Parmentier L, Olivie F, Harake R, Janssen A (2008). Injury of the axillary artery after a proximal humeral fracture: a case report and overview of the literature. *Acta Chirurgica Belgica*.

[5] Rasouli MR, Moini M, Khaji A (2009). Civilian traumatic vascular injuries of the upper extremity: report of the Iranian national trauma project. *Annals of Thoracic and Cardiovascular Surgery*.

[6] Gill H, Jenkins W, Edu S, Bekker W, Nicol AJ, Navsaria PH (2011). Civilian penetrating axillary artery injuries. *World Journal of Surgery*.

[7] Mouzopoulos G, Lassanianos N, Mouzopoulos D, Tzurbakis M, Georgilas I (2008). Axillary artery injury associated with proximal humerus fractures. *Vasa*.

[8] Byrd RG, Byrd RP, Roy TM (1998). Axillary artery injuries after proximal fracture of the humerus. *The American Journal of Emergency Medicine*.

[9] Topal AE, Eren MN (2011). Management of axillo-subclavian arterial injuries and predictors of outcome. *Minerva Chirurgica*.

[10] Testerman GM, Gonzalez GD, Dale E (2008). CT angiogram and endovascular stent graft for an axillary artery gunshot wound. *Southern Medical Journal*.

[11] Krüger A, Florido C, Braunisch A, Walther E, Yilmaz TH, Doll D (2013). Penetrating arterial trauma to the limbs: outcome of a modified protocol. *World Journal of Emergency Surgery*.

[12] Spahos T, Torella F (2012). The basilic vein: an alternative conduit for complex iliofemoral reconstruction. *European Journal of Vascular & Endovascular Surgery*.

[13] Slais M, Spacek M, Rohn V, Mitás P, Tosovský J (2007). Posterior elbow dislocation combined with subadventitial rupture of the brachial artery: interposition with the use of the autologous. *Prague Medical Report*.

[14] Lewis HG, Morrison CM, Kennedy PT, Herbert KJ (Mar 2003). Arterial reconstruction using the the zone of injury in pediatric supracondylar humeral fractures: a clinical and radiological series. *Plastic and Reconstructive Surgery*.

[15] Razif MA, Rajasingam V (2002). Anterior shoulder dislocation with artery nerve injury. *Medical Journal of Malaysia*.

